# Hypoxia-induced modulation of PTEN activity and EMT phenotypes in lung cancers

**DOI:** 10.1186/s12935-016-0308-3

**Published:** 2016-04-18

**Authors:** Takashi Kohnoh, Naozumi Hashimoto, Akira Ando, Koji Sakamoto, Shinichi Miyazaki, Daisuke Aoyama, Masaaki Kusunose, Motohiro Kimura, Norihito Omote, Kazuyoshi Imaizumi, Tsutomu Kawabe, Yoshinori Hasegawa

**Affiliations:** Department of Respiratory Medicine, Nagoya University Graduate School of Medicine, 65 Tsurumai-cho, Showa-Ku, Nagoya 466-8550 Japan; Department of Respiratory Medicine and Allergy, Fujita Health University, Toyoake, Japan; Department of Pathophysiological Laboratory Sciences, Nagoya University Graduate School of Medicine, Nagoya, Japan

**Keywords:** Lung cancer, Hypoxia, Epithelial-mesenchymal transition, β-catenin, PTEN

## Abstract

**Background:**

Persistent hypoxia stimulation, one of the most critical microenvironmental factors, accelerates the acquisition of epithelial–mesenchymal transition (EMT) phenotypes in lung cancer cells. Loss of phosphatase and tensin homologue deleted from chromosome 10 (PTEN) expression might accelerate the development of lung cancer in vivo. Recent studies suggest that tumor microenvironmental factors might modulate the PTEN activity though a decrease in total PTEN expression and an increase in phosphorylation of the PTEN C-terminus (p-PTEN), resulting in the acquisition of the EMT phenotypes. Nevertheless, it is not known whether persistent hypoxia can modulate PTEN phosphatase activity or whether hypoxia-induced EMT phenotypes are negatively regulated by the PTEN phosphatase activity. We aimed to investigate hypoxia-induced modulation of PTEN activity and EMT phenotypes in lung cancers.

**Methods:**

Western blotting was performed in five lung cancer cell lines to evaluate total PTEN expression levels and the PTEN activation. In a xenograft model of lung cancer cells with endogenous PTEN expression, the PTEN expression was evaluated by immunohistochemistry. To examine the effect of hypoxia on phenotypic alterations in lung cancer cells in vitro, the cells were cultured under hypoxia. The effect of unphosphorylated PTEN (PTEN4A) induction on hypoxia-induced EMT phenotypes was evaluated, by using a Dox-dependent gene expression system.

**Results:**

Lung cancer cells involving the EMT phenotypes showed a decrease in total PTEN expression and an increase in p-PTEN. In a xenograft model, loss of PTEN expression was observed in the tumor lesions showing tissue hypoxia. Persistent hypoxia yielded an approximately eight-fold increase in the p-PTEN/PTEN ratio in vitro. PTEN4A did not affect stabilization of hypoxia-inducible factor 1α. PTEN4A blunted hypoxia-induced EMT via inhibition of β-catenin translocation into the cytoplasm and nucleus.

**Conclusion:**

Our study strengthens the therapeutic possibility that compensatory induction of unphosphorylated PTEN may inhibit the acquisition of EMT phenotypes in lung cancer cells under persistent hypoxia.

## Background

The tumor microenvironment, which involves activation of various signal pathways, accelerates the acquisition of epithelial-mesenchymal transition (EMT) in lung cancers cells [1, 2]. Persistent hypoxia induces acquisition of EMT phenotypes, involving de novo EMT-related gene expression, such as twist, and the stabilization of hypoxia-inducible factor 1α (HIF-1α), a significant transcriptional factors in hypoxia [3–7]. As a result, persistent hypoxia stimulation has been proposed as one of the most critical microenvironmental factors in the development of cancer and tissue fibrosis in vivo [5, 6, 8, 9]. The tumor suppressor gene, phosphatase and tensin homologue deleted from chromosome 10 (PTEN), negatively regulates the activation of various signaling pathways in the tumor microenvironment [10], but hyperactivation of these pathways is often observed in lung cancers [11–13]. Loss of PTEN expression might accelerate the development of lung cancer in vivo [14]. Meanwhile, recent studies suggest that phosphorylation of the PTEN C-terminus (p-PTEN) might directly induce a loss of PTEN activity [15, 16]. We recently showed that transforming growth factor β (TGFβ), one of another inducers of EMT, might lead to loss of PTEN activity through a decrease in total PTEN expression level and TGFβ-induced phosphorylation of its C-terminus in lung cancer cells [17]. Nevertheless, it is not known whether or not persistent hypoxia might modulate the PTEN phosphatase activity in lung cancers. Furthermore, whether hypoxia-induced EMT phenotypes are negatively regulated by unphosphorylated PTEN remains elusive.

Based on the knowledge that persistent hypoxia-induced aberrant signaling pathway should be therapeutic target for lung cancer [18, 19], we aimed to determine that persistent hypoxia can modulate the PTEN activity in lung cancers and that unphosphorylated PTEN can inhibit hypoxia-induced EMT.

## Results

### Effect of persistent hypoxia on total PTEN expression and phosphorylation of the C-terminal tail in PTEN in lung cancers

**Fig. 1:**
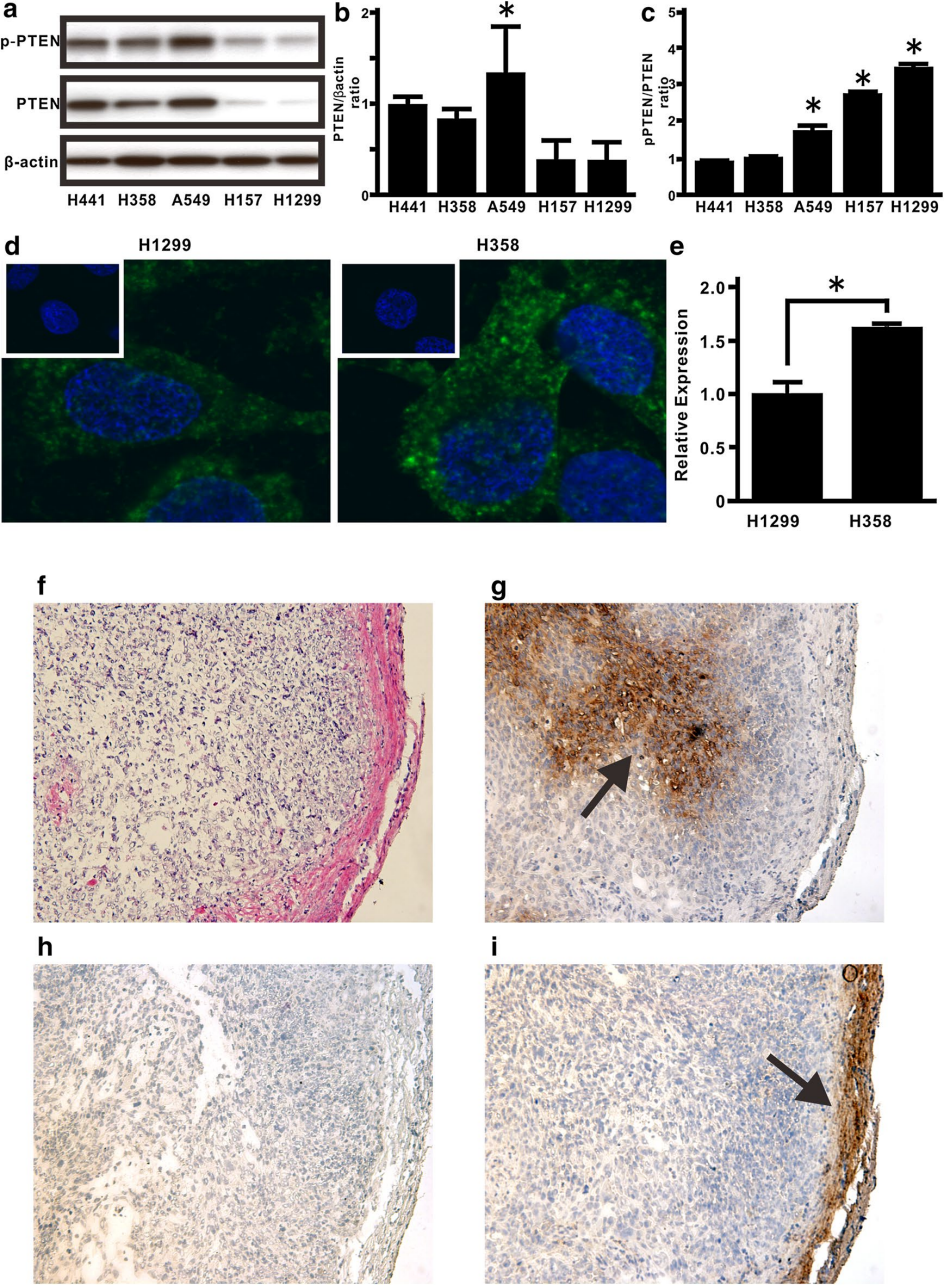
Total PTEN levels and phosphorylation levels of the PTEN C-terminus in lung cancer cells. The levels of total PTEN and p-PTEN expression were evaluated in five lung cancer cell lines. **a** Cell extracts were harvested for the analysis of p-PTEN, PTEN, and β-actin by western blotting. Equal amounts (20 μg) of protein were loaded for analysis. The ratio of total PTEN protein to β-actin, **b** and p-PTEN to total PTEN protein, **c** is presented as an intensity level relative to that in H441 cells. A representative blot is shown from three independent experiments. *Asterisk* indicates p < 0.05. (**b**, **c**), **d** Immunostaining for PTEN (*green*) was performed in H1299 (*left*) and H358 (*right*) cells. Nuclear staining was performed by Hoechst33342 (*blue*). Results are representative of three independent experiments. All images shown are × 200 magnification. **e** NIS-Elements AR software was used to compare the intensity level of PTEN expression between H1299 (*left* panels in **d**) and H358 (*right* panels in **d**) cells, compensated by cell numbers, which were counted by Hoechst33342 staining. Data represent the mean ± SE of two independent experiments. *Asterisk* indicates p < 0.05. To evaluate whether persistent hypoxia might modulate PTEN expression in lung cancer cells in vivo, we examined the tumor sections from Dox-treated nude mice at day 28 after inoculation with H358ON cells carrying GFP. **f** H–E staining was performed on tumor tissues. **g** Immunostaining for pimonidazole as a hypoxia marker was performed on a serial section of tumor tissues. An *arrow* indicates the cells positive for pimonidazole. **h** Control immunostaining for PTEN on a serial section of tumor samples is shown. **i** Immunostaining for PTEN was also performed on the serial section of tumor samples. An *arrow* indicates the cells positive for PTEN. Representative tumor sections are shown from three independent experiments. **f**–**i** All images shown are ×200 magnification

To evaluate total PTEN expression levels and the p-PTEN/PTEN ratio in lung cancer cells, western blotting was performed in the following five lung cancer cell lines: H441, H358, A549, H157 and H1299 [20]. Western blotting analysis showed that H1299 cells involving the EMT phenotypes (20), had lower PTEN expression level than H441 and H358 cells; as consequence the p-PTEN/PTEN ratio was about threefold higher in H1299 cells than in H441 and H358 cells (Fig. 1a–c). These findings were supported by immunofluorescence images showing that PTEN protein was expressed in H358 cells, whereas H1299 cells showed low PTEN expression (Fig. 1d, e). To determine whether persistent hypoxia might modulate PTEN expression in lung cancer cells in vivo, we examined the H358ON cells expressing GFP that had been grown in the flank of Dox-treated nude mice after administration of pimonidazole, which detects hypoxia via immunohistochemical staining [6, 17]. H–E staining showed tumor growth in the subcutaneous lesion of nude mice (Fig. 1f). To detect tissue hypoxia in the tumor specimen in vivo, immunostaining for pimonidazole was performed (Fig. 1g). The tumor cells stained positive for intracellular pimonidazole, indicating that tissue hypoxia might be involved in the tumor lesions. Furthermore, to evaluate the association between tissue hypoxia and PTEN expression in tumor lesions in vivo, immunostaining for PTEN in a serial section of the tumor sample was undertaken. Although the immunostaining showed no or little PTEN expression in the tumor lesions (Fig. 1h, i), positive staining for PTEN was observed in the subcutaneous tissue of nude mice (Fig. 1h, i).

### Effect of persistent hypoxia on modulation of PTEN expression and EMT phenotypes in H358 cells in vitro

**Fig. 2:**
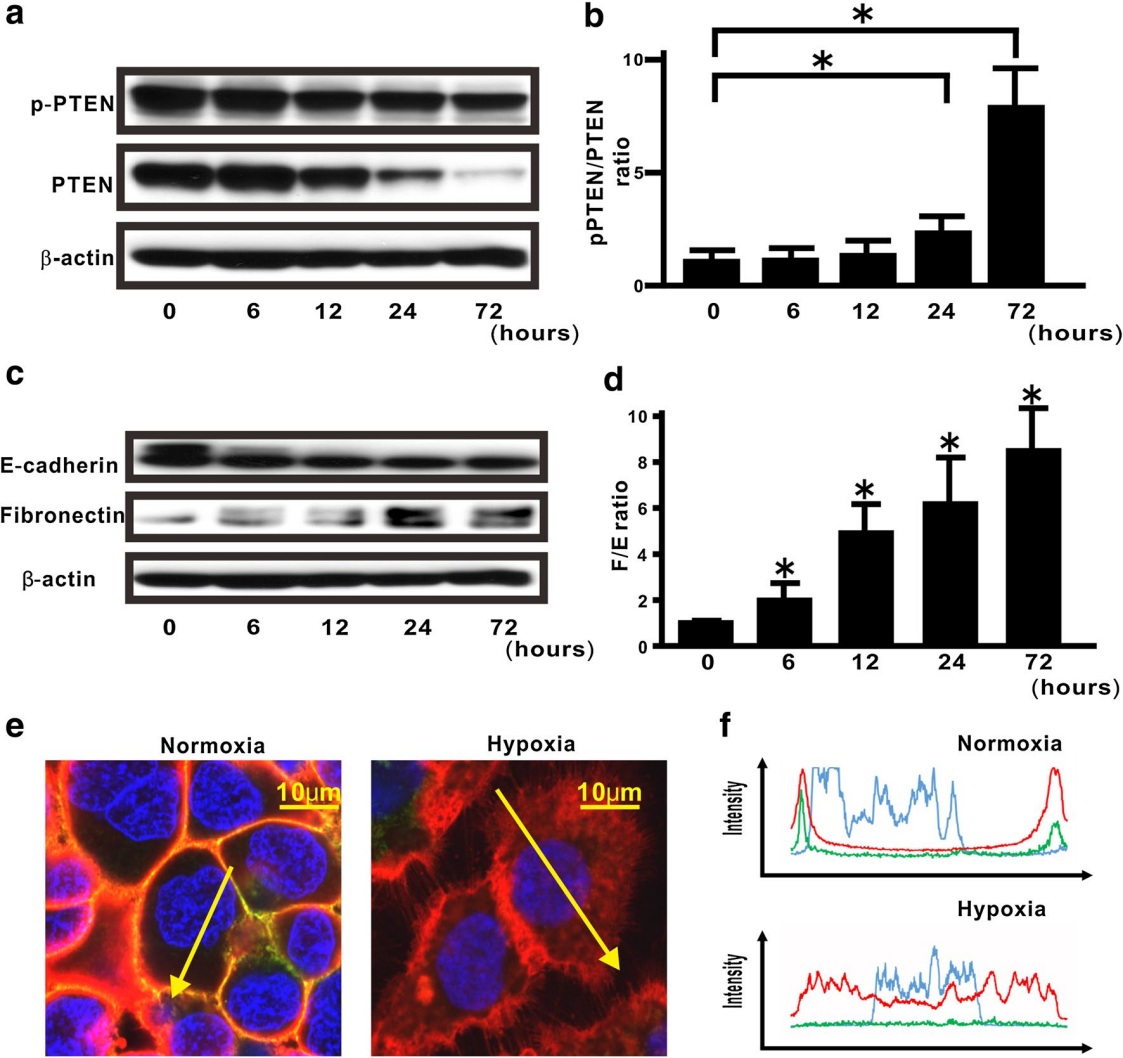
Hypoxia-induced modulation of PTEN expression and EMT in H358 cells. **a** Cell extracts were analyzed for the levels of total and phosphorylated PTEN at the indicated time. A blot is representative of three independent experiments. **b** The ratio of p-PTEN to total PTEN is presented as an intensity level relative to that in H358 naïve cells at 0 min. Data were shown as the mean ± standard error (SE). The experiment was performed three times with similar results. *Asterisk* indicates p < 0.05. **c** Western blotting analysis for fibronectin and E-cadherin at the indicated time points was carried out. A blot is representative of three independent experiments. **d** The ratio of fibronectin to E-cadherin (F/E ratio) was evaluated, compared with that in cells treated under hypoxia at 0 min (*bottom*). *Asterisk* indicates p < 0.05. **e**, **f** By using confocal laser scanning microscopy and imaging software, we evaluated the fluorescence intensities of β-catenin (*red*) and E-cadherin (*green*) in the cells under normoxia or hypoxia. Hoechst33342 (*blue*) was utilized for nuclear staining. The *left* and *right* images in **e** shows cells cultured under normoxia and hypoxia, respectively. The upper and lower panels in **f** plot the fluorescence intensity of β-catenin *red*, E-cadherin *green*, and nucleus *blue* over a cross-section of cells cultured under normoxia and hypoxia, respectively. Data are representative of at least three independent experiments

Next, to evaluate the effect of persistent hypoxia on modulation of PTEN expression in lung cancers, H358 cells were treated under hypoxia in vitro. Under persistent hypoxia stimulation, total PTEN levels decreased in a time-dependent manner but p-PTEN levels remained steady in H358 cells, leading to an approximately eightfold increase in the p-PTEN/PTEN ratio 72 h after hypoxia stimulation (Fig. 2a, b). To determine whether persistent hypoxia can induce the acquisition of EMT phenotypes in H358 cells [6], western blotting analysis for fibronectin and E-cadherin was carried out [21–23], which demonstrated that hypoxia induced EMT phenotypes in H358 cells in a time-dependent manner (Fig. 2c, d). Mounting evidence suggest that de novo expression of mesenchymal genes in epithelial cells could be induced by translocation of β-catenin into the cytoplasm and the nucleus [21, 24]. Therefore, we determined hypoxia-stimulated translocation of β-catenin in lung cancer cells. Because many studies suggest that E-cadherin is one of the most important constituents of adherens junction on cell membrane [21, 25–27], double immunostaing for β-catenin and E-cadherin was performed and then the localization was evaluated by using confocal microscopy. The results suggested that β-catenin was co-localized with E-cadherin on the cell membrane in H358 cells cultured under normoxia (Fig. 2e, f), but had translocated into the cytoplasm and the nucleus when the cells were cultured under hypoxia (Fig. 2e, f).

### Effect of unphosphorylated PTEN on hypoxia-induced EMT in H358 cells

**Fig. 3:**
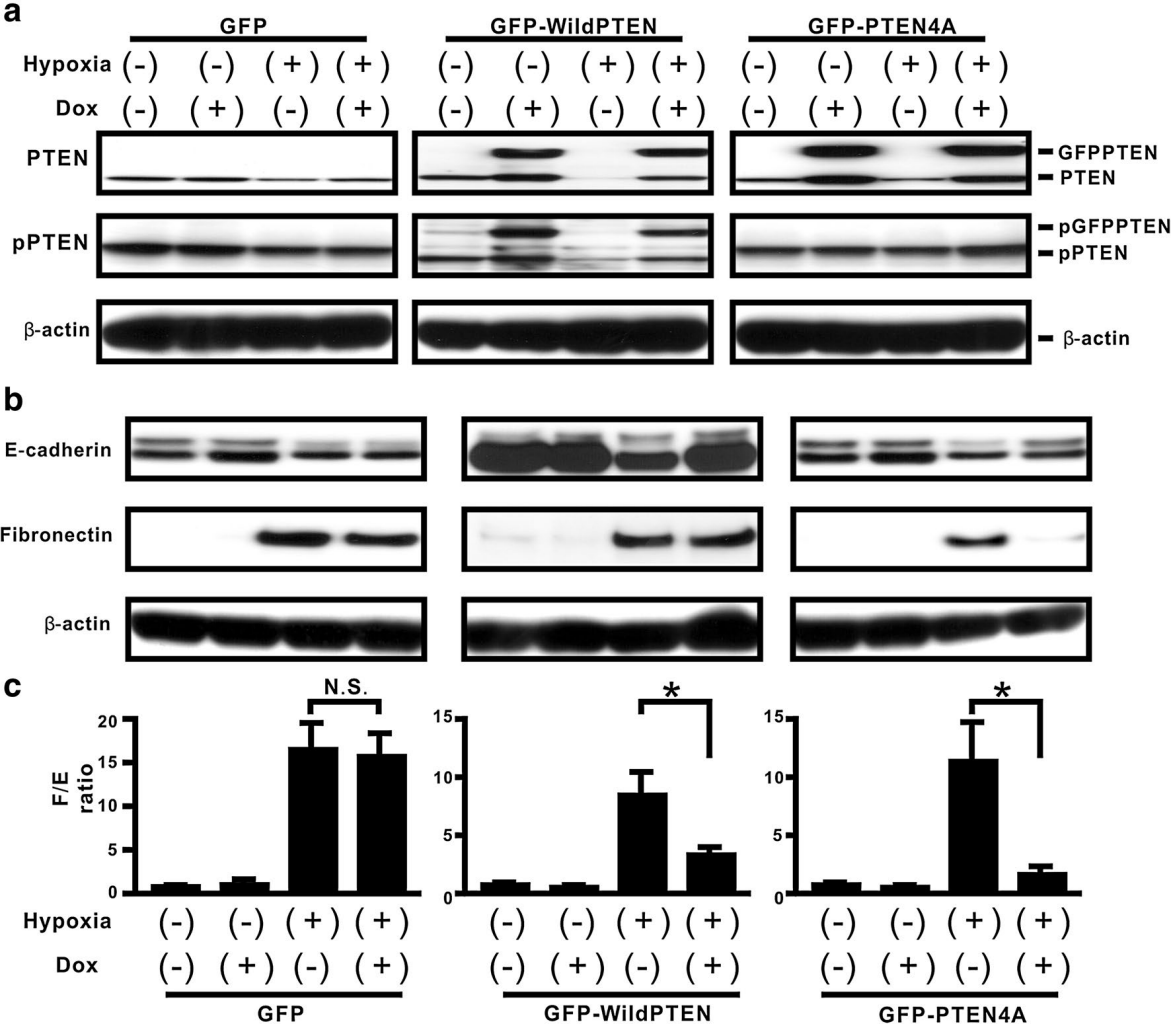
Effect of modulating phosphorylation sites in the PTEN C-terminuson hypoxia-induced EMT in H358 cells. **a** The indicated cells were treated with vehicle or Dox for 24 h before hypoxia stimulation. And then, the cells were cultured under normoxia or hypoxia for a further 24 h in the absence or presence of Dox. Western blotting analysis for total PTEN (*top*) and p-PTEN (*bottom*) was carried out. A *blot* is representative of three independent experiments (GFP, *left*; GFP-WildPTEN, *middle*; GFPPTEN4A, *right*). **b** Western blotting analysis for fibronectin and E-cadherin in the indicated cells cultured under normoxia or hypoxia for 48 h was carried out. A blot is representative of three independent experiments. **c** F/E ratio in the treated cells was compared with that in cells treated with vehicle in the absence of Dox. *Asterisk* indicates p < 0.05

We previously demonstrated that unphosphorylated PTEN inhibits TGFβ-induced acquisition of the EMT phenotypes in lung cancer cells [17]. Nevertheless, whether or not phosphorylation of the PTEN C-terminus might directly play a critical role in hypoxia-induced EMT remains elusive. Here, therefore, we evaluated the effect of unphosphorylated PTEN on hypoxia-induced EMT phenotypes, by using a Dox-dependent gene expression system (Fig. 3a). To evaluate whether hypoxia-induced EMT can be modulated by Dox-induced GFP-PTEN4A expression, western blotting analysis for fibronectin and E-cadherin was carried out. There was no or little reduction in the fibronectin/E-cadherin ratio in GFP-expressing cells; however, de novo GFP-PTEN4A protein expression by Dox yielded a significant decrease of about 88 % in the fibronectin/E-cadherin ratio (Fig. 3b, c). There was a limited repression in the fibronectin/E-cadherin ratio in GFP-WildPTEN-expressing cells (Fig. 3b, c); in particular, GFP-WildPTEN did not appear to repress hypoxia-induced fibronectin expression.

### Effect of unphosphorylated PTEN on hypoxia-induced stabilization of HIF-1α expression, translocation of β-catenin, and the EMT-related gene twist expression in H358 cells

**Fig. 4:**
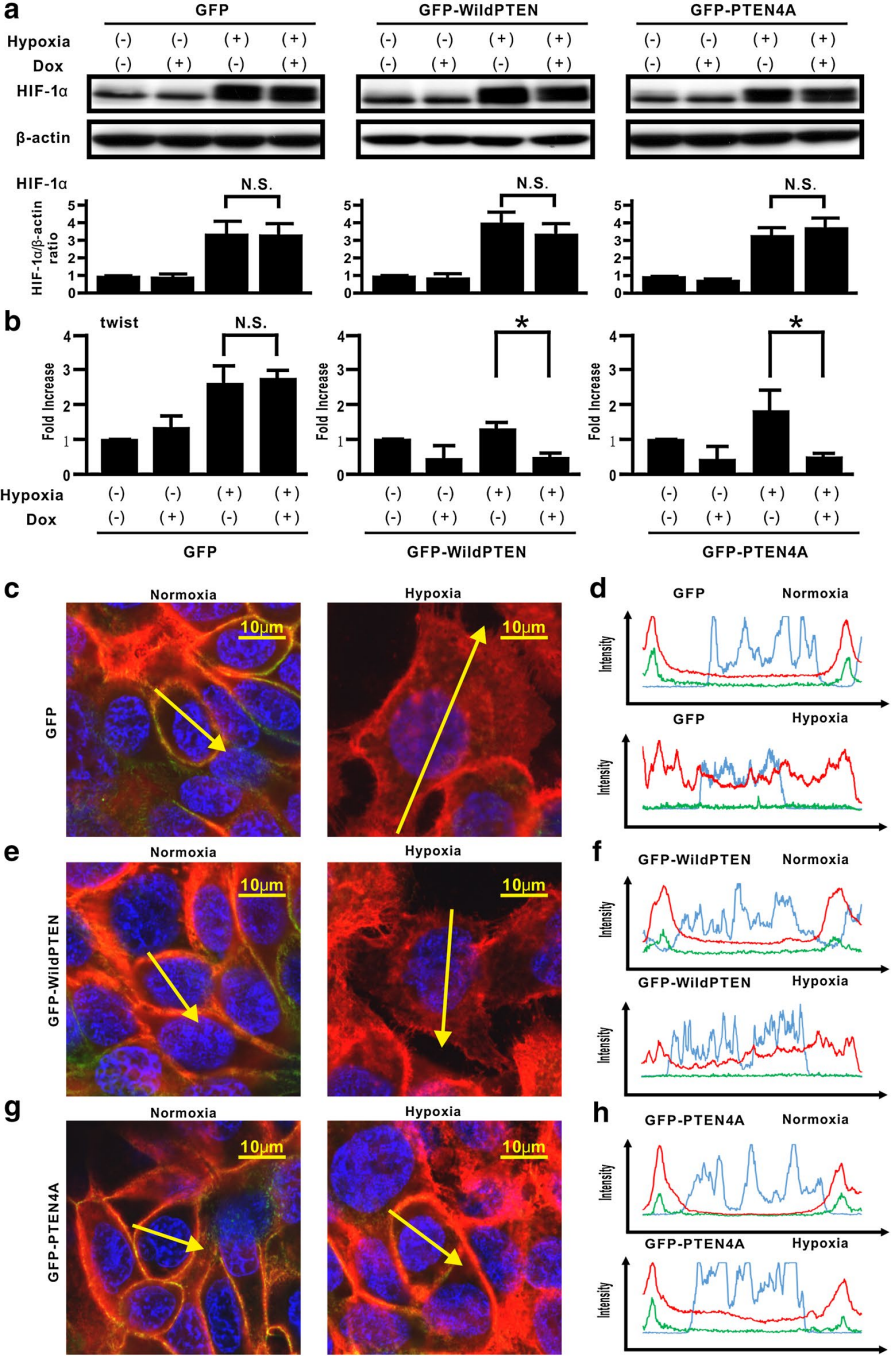
Effect of unphosphorylated PTEN on hypoxia-induced stabilization of HIF-1α expression, translocation of β-catenin, and the EMT-related gene twist expression in H358 cells. **a** The indicated cells were treated with vehicle or Dox for 24 h before hypoxia stimulation. And then, the cells were cultured under normoxia or hypoxia for a further 6 h in the absence or presence of Dox. Western blotting analysis for f HIF-1α was carried out. A blot is representative of three independent experiments (GFP, *left*; GFP-WildPTEN, *middle*; GFPPTEN4A, *right*). The ratio of HIF-1α to β-actin was compared with that in the cells cultured under normoxia in the absence of Dox. *p < 0.05 *NS* not significant. **b** The indicated cells were treated with vehicle or Dox for 24 h before hypoxia stimulation. And then, the cells were cultured under normoxia or hypoxia for a further 24 h in the absence or presence of Dox. The expression levels of twist mRNA were analyzed by using real-time PCR and normalized to GAPDH mRNA (GFP, *left*; GFP-WildPTEN, *middle*; GFPPTEN4A, *right*). The relative twist expressions were compared with that in cells treated under normoxia. Data represent the mean ± standard error of the mean (SEM) from three independent experiments. *p < 0.05, *NS* not significant. **c**–**h** The indicated cells were treated with vehicle or Dox for 24 h before hypoxia stimulation. And then, the cells were cultured under normoxia or hypoxia for a further 24 h in the absence or presence of Dox. The intensity of fluorescence of β-catenin and E-cadherin was evaluated in H358ON cells with Dox-dependent GFP (**c**, **d**), GFP-WildPTEN (**e**, **f**) or GFP-PTEN4A (**g**, **h**) was evaluated. The *left* and *right* images in **c**, **e**, **g** show cells under normoxia and hypoxia, respectively. The upper and lower panels in **d**, **f**, **h** plot the fluorescence intensity of β-catenin (*red*), E-cadherin (*green*), and nucleus (*blue*) over a cross section of cells under normoxia and hypoxia, respectively. Data are representative of at least three independent experiments

To investigate the molecular mechanisms underlying PTEN4A activity, whether hypoxia-induced signaling pathways could be modulated by GFP-PTEN4A was evaluated. A recent study demonstrated that HIF-1α expression directly regulates de novo EMT-related gene expression, causing an alteration in phenotype through EMT in a cancer metastasis model [5]. Here, Dox-induced expression of de novo GFP, GFP-WildPTEN, or GFP-PTEN4A did not repress the increase in de novo HIF-1α expression in hypoxia-stimulated H358ON cells (Fig. 4a). Next, we used real-time PCR to investigate whether the inhibitory effect of unphosphorylated PTEN on hypoxia-induced acquisition of EMT phenotypes might depend on the altered expression of EMT-related genes (Fig. 4b). Our previous study showed that hypoxia stimulation induced the increasing levels of twist, but not snail, in H358 cells [6]. In the present study, although de novo GFP expression did not alter hypoxia-induced twist expression, the increasing twist mRNA levels in hypoxia-stimulated H358ON cells expressing Dox-dependent GFP-WildPTEN and GFP-PTEN4A were repressed when Dox was added (Fig. 4b). Furthermore, we determined whether Dox-induced PTEN4A expression can modulate β-catenin translocation in hypoxia-stimulated lung cancer cells. Immunocytochemistry findings showed that translocation of β-catenin was not observed in H358ON cells expressing Dox-dependent GFP, GFP-WildPTEN, or GFP-PTEN4A when cultured under normoxia (Fig. 4c–h). Although GFP or GFP-WildPTEN protein induced by Dox did not inhibit hypoxia-induced β-catenin translocation into the cytoplasm and nucleus in H358ON cells (Fig. 4c–f), β-catenin translocation induced by hypoxia stimulation was completely inhibited by de novo GFP-PTEN4A protein (Fig. 4g, h).

## Discussion

Although PTEN mutations and deficiencies might be prevalent at the advanced tumor stage and in therapeutic resistance in many types of human cancer [12, 28–30], lung cancers rarely show mutation of the PTEN gene [12, 31]. Furthermore, mounting evidence suggests that loss of the PTEN phosphatase activity via the increasing p-PTEN/PTEN ratio might be also crucial for many types of cancer to acquire malignant phenotypes [17, 27, 32, 33]. Therefore, we first evaluated total PTEN expression level and the phosphorylation level of PTEN in lung cancer cells, by assessing five lung cancer cell lines in which the EMT phenotypes had been previously analyzed [20]. The data suggested that, among the five lung cancer cell lines, those with low PTEN expression level and a high p-PTEN/PTEN ratio showed a tendency to have a high degree of the EMT phenotypes [20]. In addition to the above findings, the importance of the tumor microenvironment was suggested by recent studies [1, 2]. Because H358 cells were suitable for evaluating the underlying mechanisms, by which tumor microenvironment factors might induce EMT [6, 17, 20, 27], we utilized H358 cells for further experiments in the present study. Evidence of persistent hypoxia has been shown in tumor implant models and pulmonary fibrosis by several reliable approaches based on the detection of hypoxia-related molecules, such as pimonidazole [6, 34, 35]. Indeed, our immunohistochemical findings in vivo suggested that tumor lesions with persistent hypoxia might show no or little PTEN expression. Because H358ON cells retain endogenous PTEN expression before inoculation into mice (Fig. 1), the decreased expression of PTEN observed in cancer cells indicates that persistent tissue hypoxia might modulate PTEN expression in vivo. It should be noted that phosphorylated PTEN expression was not detected in tumor specimens, possibly owing to our use of an antibody that is not recommended for immunohistochemistry staining. Although the acquisition of EMT phenotypes is modulated by persistent hypoxia [3–7], persistent hypoxia induces a decrease in total PTEN expression and increases the p-PTEN/PTEN ratio over time in H358 cells incubated under hypoxia. Our in vitro data suggested that persistent hypoxia markedly repressed the expression of total PTEN expression in a time-dependent manner, whereas phosphorylated PTEN levels remained persistent even after hypoxia stimulation, resulting in an approximately eightfold increase in the p-PTEN/PTEN ratio in vitro [36–38]. These findings indicate that modulation of total PTEN expression and the increasing p-PTEN/PTEN ratio might be induced not only by TGFβ activation [17] but also by persistent hypoxia stimulation in lung cancer cells. Mounting evidence show that β-catenin is located on the cell membrane via E-cadherin complexes [21, 25, 26]. We previously demonstrated that although β-catenin and E-cadherin were co-localized on the cell membrane when the cells were not treated with TGFβ, TGFβ might induce translocation of β-catenin from the cell membrane to the cytoplasm and nucleus [27]. Taken together with the previous studies [21, 25], double immunostaining for β-catenin and E-cadherin suggested that persistent hypoxia might also induce translocation of β-catenin from the cell membrane to the cytoplasm and nucleus resulting in induction of mesenchymal gene expression. Therefore, a therapeutic strategy to comprehensively regulate various factors derived from tumor microenvironment might be warranted. Nevertheless, whether or not hypoxia-induced EMT might be directly affected by the increasing phosphorylation levels of the PTEN C-terminus has not been fully evaluated. In the present study, compensatory induction of unphosphorylated PTEN blunted hypoxia-induced EMT in lung cancer cells. Although persistent hypoxia-induced HIF-1α stabilization can cause the acquisition of malignant phenotypes [5, 7], PTEN4A did not appear to modulate hypoxia-induced HIF-1α stabilization, indicating that the compensatory induction of PTEN4A inhibited the acquisition of hypoxia-induced EMT phenotypes by mechanisms other than HIF-1α stabilization [39]. Although hypoxia is known to induce de novo twist expression [5, 6], our data suggested that compensatory induction of either PTEN4A or WildPTEN induced the decreasing levels of hypoxia-induced twist gene. De novo expressed GFP-WildPTEN protein decreased the fibronectin/E-cadherin ratio and expression of twist in the cells under persistent hypoxia. Meanwhile, it did not block hypoxia-induced β-catenin translocation from the cell membrane into the cytoplasm and nucleus. A recent study suggests that fibronectin transcription is induced by β-catenin translocation from E-cadherin complexes at the cell membrane into the cytoplasm and nucleus [21]. Indeed, WildPTEN did not appear to repress hypoxia-induced fibronectin expression, whereas repression of hypoxia-induced E-cadherin was restored by induction of WildPTEN. PTEN binds E-cadherin complex via the PDZ binding domain in the PTEN C-terminus [40]. Previous studies suggested that total PTEN expression levels might affect the response to WildPTEN transduction in glioma cells [28–30]. In the present study, we showed that PTEN protein was abundantly expressed in H358 cells under normoxia, whereas persistent hypoxia induced the repression of total PTEN expression in H358 cells to the same degree as that in H1299 cells. Taken together, compensatory induction of WildPTEN might restore the repression of E-cadherin expression in hypoxia-induced H358 cells showing little expression of PTEN. Thus, exogenous administration of the PTEN gene might be hopeful for treatment of lung cancer under persistent hypoxia. It should be noted that de novo GFP-PTEN4A protein expression showed an approximately 40 % greater potential to block hypoxia-induced EMT in comparison with de novo GFP-WildPTEN protein expression. We demonstrated that unphosphorylated PTEN, but not WildPTEN, inhibits TGFβ-induced EMT [17]. Research into the role of unphosphorylated PTEN in EMT induced by tumor microenvironmental factors remains important [27].

In summary, our studies strengthen the therapeutic possibility that compensatory induction of unphosphorylated PTEN may inhibit the acquisition of EMT phenotypes in lung cancers under tissue microenvironments involving persistent hypoxia stimulation.

## Methods

### Materials

All antibodies used in our experiments were purchased from the commercial companies as follow: monoclonal mouse anti-hypoxia-inducible factor 1α (HIF-1α) antibody was from Novus biologics (Littleton, CO); monoclonal mouse anti-PTEN antibody (clone 6H2.1) was from Cascade Bioscience (Winchester, UK); anti-β-catenin antibody and purified and fluorescein isothiocyanate (FITC)-conjugated mouse anti-E-cadherin antibody were from BD Biosciences (San Diego, CA); purified anti-fibronectin antibody was from Santa Cruz Biotechnology, Inc (Santa Cruz, CA); streptavidin (SAv)-Alexa 594 (SAv-594)-conjugated and SAv-Alexa 488 (SAv-488)-conjugated anti mouse antibody was from Invitrogen Life Technologies (Carlsbad, CA); purified rabbit anti-phospho-PTEN (Ser380/Thr382/Thr383) antibody was from Cell Signaling Technology (Boston, MA); Affinity-isolated rabbit anti-actin antibody was from Sigma-Aldrich (St. Louis, MO). Hoechst33342 was from Dojindo (Kumamoto, Japan). The Hypoxyprobe-1 plus kit was purchased from Hypoxyprobe, Inc. (Burlington, MA). Horseradish peroxidase (HRP)-conjugated anti-mouse antibody, 3, 3′-diaminobenzidine (DAB) Substrate Kit, and Hematoxylin QS were from Vector Laboratories (Burlingame, CA). PhosSTOP was from Roche Applied Science (Mannheim, Germany). Can Get Signal was also purchased from Toyobo Co. (Tokyo, Japan). Doxycycline (Dox) was from Clontech (Mountain View, CA). pTRE-Tight vector and pTet-On Advanced were from Clontech (Mountain View, CA).

### Vectors and gene transfection

A Dox-dependent gene expression system was applied to H358 cells, a human lung cancer cell line, carrying pTet-On Advanced (H358ON) [17]. The H358ON cells that were generated contained GFP in the pTRE-Tight vector (GFP), GFP-PTEN in the pTRE-Tight vector (GFP-WildPTEN), or GFP-PTEN with a four-Ala substitution (S380A, T382A, T383A, and S385A) on the C-terminal tail in PTEN (PTEN4A) in the pTRE-Tight vector (GFP-PTEN4A).

### Cells

Human lung cell lines H441, H358, A549, H157 and H1299, were maintained in RPMI medium as previously described [20]. To examine the effect of hypoxia on phenotypic alterations in lung alveolar cell lines in vitro, the cells were cultured under hypoxia (1 % concentration of oxygen; 1 % O_2_) for the indicated periods using a hypoxic chamber (Wakenyaku Co. Ltd., Tokyo, Japan) for the indicated periods. For immunocytochemistry, some cells were cultured in an 8-well Lab-Tek Chamber Slide System (Nalge Nunc International, Naperville, IL). Pimonidazole hydrochloride at final concentration of 150 μM was added 90 min before immunostaining for pimonidazole [6].

### Real-time PCR

We performed real-time PCR, by using a TaqMan ABI 7300 Sequence Detection System (PE Applied Biosystems, Foster City, CA). The following oligonucleotide primers and probe were used: twist (twist1: NM_000474) sense (5′-CCAGCTATGTGGCTCACGAG-3′) and antisense (5′-CTAGTGGGACGCGGACATGG-3′), internal fluorescence-labeled probe (FAM) (5′-CCTCCATCCTCCAGACCGAGAAGGCG-3′) [20]. The twist mRNA expression levels were normalized to the glyceraldehydes-3-phophate dehydrogenase (GAPDH) mRNAs signal [41].

### Western blotting analysis

Protein immunodetection was performed by electrophoretic transfer of SDS-PAGE, separation of proteins on nitrocellulose, incubation with primary antiboby, followed by the appropriate HRP-conjugate second antibody, and chemiluminescent second-step detection using ECL Plus (GE Healthcare Bio-Sciences Corp; Piscataway, NJ) [17]. The amount of protein on the immunoblots was quantified by the image software Quantity One (BioRad Laboratories, Philadelphia, PA) [20].

### Immunocytochemistry and immunehistochemistry

Immunocytochemistry and immunohistochemistry was done as previously described [9, 42, 43]. Immunofluorescent staining for PTEN in vitro was performed for H358 cells and H1299 cells and SAv-488-conjugated anti mouse antibody was utilized to visualize antibody binding. Nuclear staining was performed by Hoechst33342. To determine the impact of PTEN4A transduction on hypoxia-induced β-catenin translocation, double immunostaining for β-catenin and E-cadherin was done for H358 cells and H358ON cells with Dox-dependent GFP, GFP-WildPTEN, or GFP-PTEN4A according the previous procedure [27]. We determined distribution of β-catenin by using confocal laser scanning microscopy (TiEA1R; Nikon Instech Co., Tokyo, Japan). Imaging software (NIS-Elements AR; Nikon Instech Co., Tokyo, Japan) was utilized for the intensity of β-catenin fluorescence. To evaluate the fluorescence, intensities over a random cross section of the cells were plotted [27]. Measurement of fluorescence intensity in the nucleus and the cytoplasm was performed by evaluation of a minimum of five randomly selected high-power fields per sample [44]. Immunostaining for pimonidazole in vivo was performed by following the manufacturer’s instructions with minor modifications [6]. To evaluate PTEN expression in the tumor specimens in vivo, a DAB Substrate Kit was used to visualize antibody binding [42]. Sections were counterstained with Hematoxylin QS.

#### Mouse tumor implantation in vivo

H358ON cells (3 × 10^6^) expressing Dox-dependent GFP were inoculated subcutaneously into the flank of 6-week-old female nude mice. The treated mice were maintained on water containing Dox at a final concentration of 2 mg/ml [17].

### Statistical analysis

Non-normally distributed results were analyzed by using the Mann–Whitney test for comparison between any two groups. Furthermore, they were analyzed by non-parametric equivalents of analysis of variance (ANOVA) for multiple comparisons. A p value of less than 0.05 was considered statistically significant.
